# Integrated noninvasive diagnostics for prediction of survival in immunotherapy

**DOI:** 10.1016/j.iotech.2024.100723

**Published:** 2024-07-09

**Authors:** M. Yeghaian, Z. Bodalal, T.M. Tareco Bucho, I. Kurilova, C.U. Blank, E.F. Smit, M.S. van der Heijden, T.D.L. Nguyen-Kim, D. van den Broek, R.G.H. Beets-Tan, S. Trebeschi

**Affiliations:** 1 Department of Radiology, The Netherlands Cancer Institute, Amsterdam, The Netherlands; 2 GROW Research Institute for Oncology and Reproduction, Maastricht University, Maastricht, The Netherlands; 3 Department of Medical Oncology, The Netherlands Cancer Institute, Amsterdam, The Netherlands; 4 Pulmonology Department, Leiden University Medical Center, Leiden, The Netherlands; 5 Department of Molecular Carcinogenesis, The Netherlands Cancer Institute, Amsterdam, The Netherlands; 6 Institute of Diagnostic and Interventional Radiology, University Hospital of Zurich, Zurich, Switzerland; 7 Department of Laboratory Medicine, The Netherlands Cancer Institute, Amsterdam, The Netherlands; 8 Faculty of Health Sciences, University of Southern Denmark, Odense, Denmark

**Keywords:** cancer survival prediction, artificial intelligence, machine learning, integrated diagnostics, immunotherapy, longitudinal data

## Abstract

**Background:**

Integrating complementary diagnostic data sources promises enhanced robustness in the predictive performance of artificial intelligence (AI) models, a crucial requirement for future clinical validation/implementation. In this study, we investigate the potential value of integrating data from noninvasive diagnostic modalities, including chest computed tomography (CT) imaging, routine laboratory blood tests, and clinical parameters, to retrospectively predict 1-year survival in a cohort of patients with advanced non-small-cell lung cancer, melanoma, and urothelial cancer treated with immunotherapy.

**Patients and methods:**

The study included 475 patients, of whom 444 had longitudinal CT scans and 475 had longitudinal laboratory data. An ensemble of AI models was trained on data from each diagnostic modality, and subsequently, a model-agnostic integration approach was adopted for combining the prediction probabilities of each modality and producing an integrated decision.

**Results:**

Integrating different diagnostic data demonstrated a modest increase in predictive performance. The highest area under the curve (AUC) was achieved by CT and laboratory data integration (AUC of 0.83, 95% confidence interval 0.81-0.85, *P* < 0.001), whereas the performance of individual models trained on laboratory and CT data independently yielded AUCs of 0.81 and 0.73, respectively.

**Conclusions:**

In our retrospective cohort, integrating different noninvasive data modalities improved performance.

## Highlights

• Patients with non-small-cell lung cancer, melanoma, and urothelial cancer treated with immunotherapy were studied.
• Longitudinal CT imaging, routine blood tests, and clinical parameters were analyzed.
• An ensemble of AI models was used for 1-year survival prediction.
• Integration of noninvasive diagnostic data improved the predictive performance.

## Introduction

With the digitization of medicine, ever-increasing volumes of data are being generated by patients during their treatment, including clinical data, tissue samples, scans of pathological slides, blood tests, and radiological imaging. These multimodal data collectively encode the patient’s baseline characteristics and changes occurring during treatment. Consequently, modern healthcare centers have inadvertently become data repositories for expansive medical data. The field of medical artificial intelligence (AI) has emerged to harness these large stores of patient data and help address open needs/questions in the clinics/research field.

A prominent open need exists in the domain of immunotherapy where, despite the revolutionary advances in immune checkpoint inhibitors over the past decade, there is not yet a single standardized tissue or blood-based biomarker for the effective selection of good therapeutic candidates.^1^ Effective patient stratification/selection would prevent unnecessary exposure to ineffective therapy, mitigate the risk of side-effects for the patient, and save resources for the healthcare center.^1^^,^^2^ Previous literature has extensively explored the potential of harnessing AI methods to unlock predictive and prognostic information for immunotherapy from computed tomography (CT) imaging,^3^, ^4^, ^5^ digital pathology imaging,^6^ genomic,^7^ and transcriptomic data.^8^

In routine practice, healthcare practitioners make use of a combination of the available information for optimal patient treatment stratification and response assessment.^9^ Just as humans perform better given more contextual information, the field of multimodal data integration is built on the driving hypothesis that data from diverse sources can potentially contain complementary information, enhancing the performance of predictive models.

Multimodal data integration has yielded significant improvements in the predictive performance of AI models in other fields of research, for example, autonomous driving and video classification.^9^, ^10^, ^11^ Based on these early successes, integrative approaches were also applied to the field of medical AI,^9^ particularly in oncology.^12^ Integration of different high-dimensional omics data, characterizing cancer on different levels, has been widely explored in literature,^13^, ^14^, ^15^, ^16^ particularly given the availability of large public multimodal datasets of molecular and histopathological data from The Cancer Genome Atlas (TCGA).

Combining multiple noninvasive sources of clinical data, routinely acquired in large amounts during patient treatment and follow-up, could potentially be a promising step for precision medicine.^9^^,^^17^^,^^18^ In this study, we investigated the potential benefits of integrating imaging (CT), blood-based laboratory markers, and a few clinical parameters to predict 1-year survival in a longitudinal, retrospective cohort of patients with metastatic cancer [non-small-cell lung cancer (NSCLC), melanoma, and urothelial cancer] treated with immune checkpoint inhibition.

## Materials and methods

### Study cohort

We included a retrospective cohort of patients with stage IV melanoma, NSCLC, and urothelial cancer who were treated with anti-programmed cell death protein 1 (PD-1)/programmed death-ligand 1 (PD-L1) immune checkpoint blockade as monotherapy at our institution between 2014 and 2016. Patient characteristics are provided in Table 1. Radiological follow-up was carried out using contrast-enhanced CT, with follow-up intervals of 8-12 weeks. Besides CT imaging, data on blood-based routine laboratory tests were retrieved. We included data on all available pretreatment and on-treatment examinations (CT scans and/or laboratory tests) acquired between 3 months before the start of the treatment and up to 1 year after. Imaging and laboratory tests were abundant at different frequencies along the treatment timeline, therefore, they were paired based on closeness in acquisition date [median 4 (interquartile range 0-7)] days, allowing a maximum of a 2-month interval between the two modalities in each pair when necessary. Clinical parameters of age, sex, and tumor type were also retrieved for all patients at the start of the treatment. Death dates of patients were acquired when applicable, and the survival prediction is formulated as a binary classification task to predict survival 1 year after the examination acquisition date. This dataset represents a longitudinal and multimodal expansion of the datasets previously described in ^3^^,^^4^^,^^19^

**Table 1:** Cohort characteristics

Characteristics	Values
Age (years), median (range)	63 (28-93)
Sex, *n* (%)	
Male	286 (60)
Female	189 (40)
Cancer type, *n* (%)	
NSCLC	171 (36)
Melanoma	207 (44)
Urothelial cancer	97 (20)
Treatment, *n* (%)	
Nivolumab	475 (100)
Outcome, *n* (%)	
Death	361 (76)

NSCLC, non-small-cell lung cancer.

### Data preprocessing, model training, and validation

All the CT scans were cropped to only include the thoracic region using the method proposed by Zhang et al.^20^ The scans were then resampled into 2-mm isotropic voxel size and standardized. Missing laboratory data were discarded and/or imputed with a multivariate iterative imputer with the Bayesian Ridge regression estimator,^21^, ^22^, ^23^, ^24^ as described in more detail in Supplementary Material and Supplementary Table S7, available at https://doi.org/10.1016/j.iotech.2024.100723.

AI models were used to predict the 1-year survival of patients in a supervised manner. 3D ResNet18-like^25^ convolutional neural networks (CNNs) were trained with chest CT scans, random forest (RF)^26^ models were trained with 33 laboratory parameters (listed in Supplementary Table S1, available at https://doi.org/10.1016/j.iotech.2024.100723), and support vector machines (SVMs)^27^ were trained with three nonlongitudinal clinical parameters. All available longitudinal, pre and on-treatment, examinations in the train sets were used for training the AI models. To incorporate a temporal dimension into the models and distinguish the utilized longitudinal examinations along the treatment timeline, the intervals between the acquisition of the examination and the start of the treatment (in days) were also included as additional input features in the longitudinal modalities. These intervals were represented as normalized scalar values reflecting the position of the examination on the treatment timeline.

A total of 30 splits of Monte Carlo cross-validation (MCCV) were used for the training and validation of the AI models (Supplementary Figure S1, available at https://doi.org/10.1016/j.iotech.2024.100723)^28^ The data were split on a patient basis, with each patient having variable numbers of examinations. At each MCCV split, 26% of patients having paired CT imaging and laboratory measurements were randomly allocated for testing. The remaining patients were randomly used to train and test modality-specific AI models, (train set: 80%, train hold-out set: 20%). Validation was carried out using the entire dataset, as well as various subsets grouped by early and late treatment stages, and by cancer type. Moreover, we utilized Shapely Additive exPlanation (SHAP) to explain the decisions made by the RF model.^29^

Scikit-learn 0.24.1,^30^ Keras 2.2.4,^31^ and Tensorflow-gpu 1.12^32^ were used for the implementation of the models. Further details regarding preprocessing and model training are provided in Supplementary Material, available at https://doi.org/10.1016/j.iotech.2024.100723.

### Multimodal integration strategy for survival prediction

**Figure 1:**
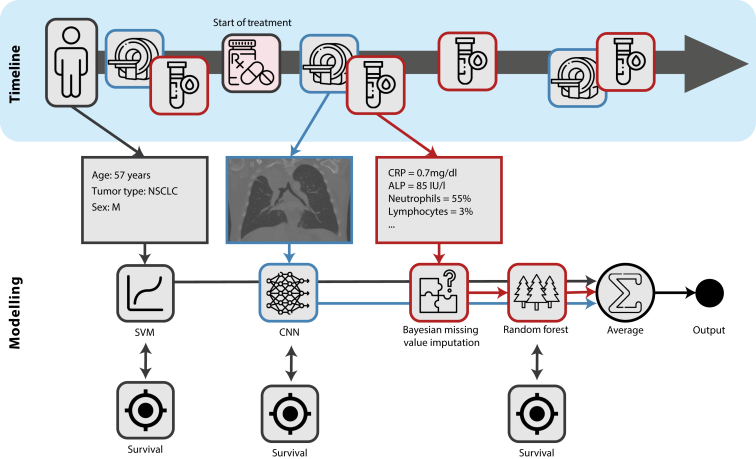
**Schematic representation of the model-agnostic late fusion integration strategy.** ALP, alkaline phosphatase; CNN, convolutional neural network; CRP, C-reactive protein; NSCLC, non-small cell lung cancer; SVM, support vector machine.

Model-agnostic decision-based late fusion strategy^10^^,^^33^ was adopted to integrate CT imaging, laboratory, and clinical data: prediction probabilities of the single-modality classifiers were first computed independently, then aggregated by averaging (Figure 1). Only matching patient data were used during testing. This approach allows the individual training of medical datasets with the presence of missing modalities or not-aligned modalities. It also handles missing modalities at test time.

### Statistical analysis

Prognostic performance was evaluated using the area under the receiver operating curve (ROC-AUC). Sensitivity and specificity scores were also calculated to support the AUC. Confidence intervals were calculated using 1000-times bootstrapping via repeated sampling with replacement. The statistical significance of the classifiers was tested using the Mann–Whitney *U* test. McNemar’s test was further used to compare the differences between the classifications of different combinations of modalities. The statistical significance of the changes in the AUCs of different subsets of the longitudinal data was tested using the Hanley and McNeil method. Clinical significance was calculated with Kaplan–Meier survival curves, the log-rank test, and the difference in median survival time between the two groups. A *P* value <0.05 was considered statistically significant. Additional supporting metrics, including sensitivity, specificity, positive predictive value, and negative predictive value, were also added at the median threshold.

## Results

### Study cohort

We included 475 patients treated at the Netherlands Cancer Institute - AVL Hospital, Amsterdam, between 2014 and 2016 with immunotherapy (anti-PD-L1 or anti-PD-1 immune checkpoint inhibition). Among them, 207 were patients with stage IV melanoma, 171 were patients with stage IV NSCLC, and 97 were patients with stage IV urothelial cancer. A total of 444 patients had CT imaging, 475 had blood-based laboratory data, and 444 had both imaging and laboratory data (Table 1 and Supplementary Figure S2, available at https://doi.org/10.1016/j.iotech.2024.100723). All patients had clinical parameters of age, sex, and type of cancer. Overall, 1702 longitudinal CT scans and 7919 longitudinal laboratory examinations were used in the analysis for training and testing.

### Individual modalities

**Figure 2:**
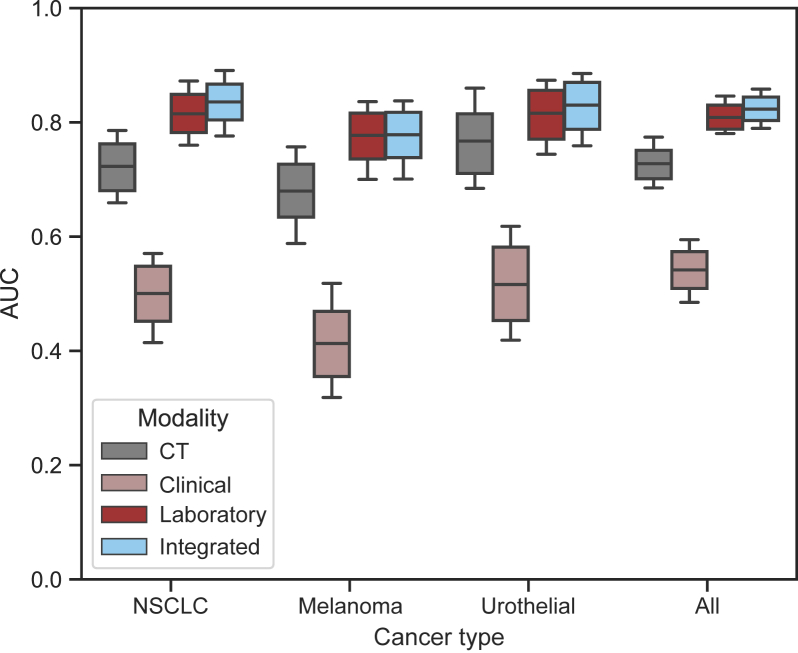
**Survival classification performance evaluation based on cancer type using individual and integrated modalities.** AUC, area under the curve; CT, computed tomography; NSCLC, non-small-cell lung cancer.

The prognostic performance of individual diagnostic modalities was tested using a total of 1559 unique examinations across all 30 MCCV test splits of random 115 patients. Overall, across the entire patient treatment timeline, blood-based laboratory data showed higher prognostic value than imaging (AUC = 0.81 versus 0.73), followed by clinical data (AUC = 0.54; Table 2 and Figure 2). In general, pretreatment showed the lowest results, with the largest decrease observed in blood values (AUC = 0.69), followed by CT scans (AUC = 0.66). The highest performance was observed 6-9 months during treatment to predict whether the patient would be alive 1 year after (AUC_blood_ = 0.88, AUC_CT_ = 0.75; Table 3 and Supplementary Figure S5, available at https://doi.org/10.1016/j.iotech.2024.100723). Clinical data were not longitudinal to be dissected across different time points. In terms of cancer type, the highest performance was observed for patients with urothelial cancer (AUC_CT_ = 0.77, AUC_blood_ = 0.82) followed by those with NSCLC (AUC_CT_ = 0.72, AUC_blood_ = 0.81) and melanoma (AUC_CT_ = 0.68, AUC_blood_ = 0.78; Table 4 and Figure 2). In terms of survival, blood values showed a survival difference between high- and low-risk groups (split on median) of 133 days for pretreatment data and 227 and 88 days for on-treatment at 3 and 6 months, respectively. Similarly, CT showed 123 days for pretreatment data and 206 and 99 days for on-treatment at 3 and 6 months, respectively (Supplementary Table S5, available at https://doi.org/10.1016/j.iotech.2024.100723).

**Table 2:** The prognostic performance of individual and integrated modalities

Modality	(*n* survival, *n* death)	AUC (95% CI)	Sensitivity (95% CI)	Specificity (95% CI)	Positive predictive value (95% CI)	Negative predictive value (95% CI)	*P* value
CT	(942, 617)	0.73 (0.70-0.75)	0.60 (0.57-0.63)	0.75 (0.71-0.78)	0.79 (0.75-0.81)	0.55 (0.52-0.58)	<0.001
Laboratory	(942, 617)	0.81 (0.79-0.83)	0.68 (0.65-0.71)	0.78 (0.75-0.81)	0.83 (0.80-0.85)	0.61 (0.58-0.65)	<0.001
Clinical	(1099, 460)	0.54 (0.51-0.57)	0.52 (0.49-0.55)	0.55 (0.50-0.59)	0.73 (0.70-0.77)	0.32 (0.29-0.36)	0.010
Integrated longitudinal	(942, 617)	**0.83 (0.81-0.85)**	0.67 (0.64-0.70)	0.79 (0.76-0.82)	0.83 (0.81-0.86)	0.61 (0.58-0.64)	<0.001
Integrated all	(942, 617)	0.82 (0.80-0.84)	**0.68 (0.65-0.70)**	**0.80 (0.77-0.83)**	**0.84 (0.81-0.86)**	**0.62 (0.58-0.65)**	<0.001

*n* indicates the number of examinations.

AUC, area under the curve; CI, confidence interval; CT, computed tomography.

**Table 3:** The prognostic performance of pretreatment and on-treatment longitudinal data modalities in 3-month intervals, using the latest examination (pair) per patient in each interval

	Modality	Pretreatment	On-treatment			
		-92-0 days	0-92 days	92-184 days	184-276 days	276-365 days
AUC (95% CI)	CT	0.64 (0.59-0.70)	0.73 (0.67-0.78)	0.78 (0.72-0.84)	0.75 (0.66-0.84)	0.74 (0.62-0.84)
	Laboratory	0.70 (0.65-0.75)	0.81 (0.76-0.85)	0.83 (0.78-0.89)	0.88 (0.81-0.94)	0.83 (0.74-0.90)
	Integrated	**0.71 (0.66-0.76)**	**0.83 (0.78-0.87)**	**0.86 (0.81-0.91)**	**0.89 (0.81-0.94)**	**0.84 (0.75-0.91)**
*P* value	All experiments	<0.001	<0.001	<0.001	<0.001	<0.001

The days in the intervals are relative to start of treatment (SoT). For example, the pretreatment interval (-92 to 0 days) refers to the duration from 3 months before SoT up to SoT. The highest results, which correspond to the integrated modalities, are highlighted in bold.

AUC, area under the curve; CI, confidence interval; CT, computed tomography.

**Table 4:** The prognostic performance of individual and integrated modalities stratified by cancer type

Modality	AUC (95% confidence interval)			
	NSCLC	Melanoma	Urothelial	All combined
CT	0.72 (0.68-0.76)	0.68 (0.63-0.73)	0.77 (0.71-0.81)	0.73 (0.70-0.75)
Laboratory	0.81 (0.78-0.85)	0.78 (0.74-0.82)	0.82 (0.77-0.86)	0.81 (0.79-0.83)
Clinical	0.50^a^ (0.45-0.55)	0.41 (0.36-0.47)	0.52^b^ (0.45-0.58)	0.54 (0.51-0.57)
**Integrated**	**0.84 (0.80-0.87)**	**0.78 (0.74-0.82)**	**0.83 (0.79-0.87)**	**0.82 (0.80-0.85)**

*P* < 0.05 in all experiments, except the ones marked with an ^a^ and ^b^, in which *P* = 0.997 and *P* = 0.563, respectively. The highest results, which correspond to the integrated modalities, are highlighted in bold.

AUC, area under the curve; CT, computed tomography; NSCLC, non-small-cell lung cancer.

### Integrated modalities

The prognostic performance of the integrated diagnostic modalities was tested with the same endpoints as in the single modalities. Across all endpoints and subanalysis, the integrated scheme was equal to or exceeded the performance of each single modality: reaching an AUC of 0.83 versus 0.81 of the best-performing single modality across the entire treatment timeline, 0.71 versus 0.70 of the best-performing single modality on pretreatment examinations, and 0.89 versus 0.88 on 6-9 months on-treatment examinations. The largest increase was observed in the first 6 months of treatment, from 0.83 to 0.86. A similar trend was observed in individual cancer types, with the largest increase in NSCLC from 0.81 to 0.84 AUC. In terms of survival, integrated data showed higher performance, with the largest increase observed in the first 3 months of treatment, where high- and low-risk patients stratified according to integrated data showed a difference of 239 days. Figure 2 shows a schematic of the overall performance, across cancer types and modalities. (Supplementary Figures S3-S5, available at https://doi.org/10.1016/j.iotech.2024.100723). show the performance of all combinations of modalities, and Kaplan-Meier survival curves. More detailed results can also be found in Table 2, Table 3, Table 4 and Supplementary Tables S2-S6, available at https://doi.org/10.1016/j.iotech.2024.100723.

### Explainability of AI models

**Figure 3:**
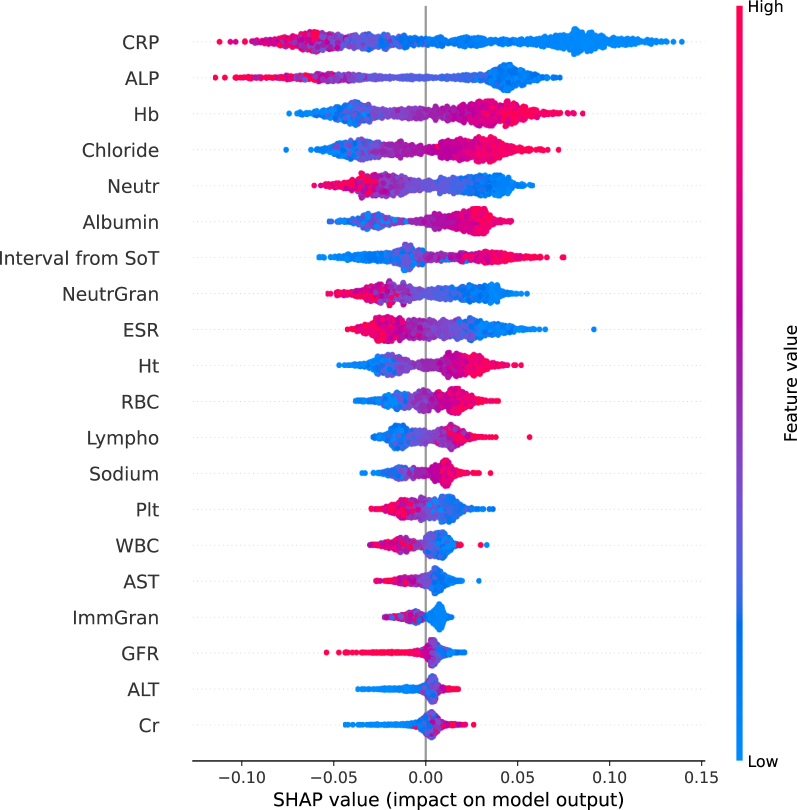
**Interpretability of the random forest model trained with laboratory data using Shapely Additive exPlanation (SHAP).** ALP, alkaline phosphatase; ALT, alanine aminotransferase; AST, aspartate aminotransferase; CNN, convolutional neural network; Cr, creatinine; CRP, C-reactive protein; ESR, erythrocyte sedimentation rate; GFR, glomerular filtration rate; Hb, hemoglobin; Ht, hematocrit; ImmGran, immature granulocytes; Lympho, lymphocytes; Neutr, neutrophils; NeutrGran, a combination of neutrophils, basophils and eosinophils; Plt, platelets; RBC, red blood cell; SoT, start of treatment; WBC, white blood cell.

For the RF model, average SHAP values were calculated for all the unique laboratory examinations across all MCCV test splits (*n* = 1559). The features with the highest average impact on the output of the model are presented in Figure 3, which are ordered by their average importance for the task of survival prediction. SHAP explanations showed that the CRP feature, reflecting the serum C-reactive protein level, was found, on average, to be the most impactful feature on the prediction of the likelihood of patient survival. Lower values of serum CRP (blue) were positively correlated with survival. Therefore according to the model’s explanation, patients with lower values of CRP were more likely to survive. Similarly, lower levels of alkaline phosphatase (ALP), which was the second most important feature, showed a positive correlation with survival prediction. The third important feature was shown to be hemoglobin (Hb), with higher levels of Hb contributing to the model’s prediction of survival likelihood. SHAP summary plots were also generated for each tumor type within our patient cohort (Supplementary Figures S6-S8, available at https://doi.org/10.1016/j.iotech.2024.100723), where the same/very similar features were shown to be important.

## Discussion

Our aim was to investigate the potential value that noninvasive integrated diagnostics could bring to the prediction of 1-year survival in patients with NSCLC, melanoma, and urothelial cancer treated with immunotherapy. We focused on integrating longitudinal radiological CT imaging with laboratory blood data, alongside several clinical parameters. CT imaging was included to capture anatomical characteristics that could potentially aid in survival prediction. Blood-based laboratory data also provide insight into the general health of the patient (and potentially inflammation-related information). Each data modality was trained individually in a supervised manner using specific AI models: CNNs for CT imaging, RFs for laboratory data, and SVMs for clinical data. An ensemble of models was trained for each data modality using MCCV. In our study, we trained the models to predict the probability of 1-year survival from any given time point. For example, if the input was data 3 months into treatment, survival was predicted 1 year and 3 months after the start of treatment. The prediction probabilities of each modality were then aggregated into a final integrated decision.

As a single modality, laboratory data had the greatest predictive performance. This finding could be due to the predictive power of blood markers but it could also be influenced by the frequency of acquisition of blood tests, which leads to the availability of larger amounts of longitudinal laboratory data for training the AI models compared with other modalities. Combined with the other modalities, there was a modest but consistent and statistically significant improvement (McNemar’s test, *P* = 0.04) in the predictive performance of the AI model trained only with laboratory data. Whether trained on a single modality or using an integrated approach, AI-based predictions showed significant discriminative ability between high-risk and low-risk groups on Kaplan–Meier survival curves for pretreatment data and on-treatment data at 3 and 6 months (Supplementary Figure S5 and Table S5, available at https://doi.org/10.1016/j.iotech.2024.100723).

SHAP values showed that, on average, the most impactful feature on the model predictions was the C-reactive protein (CRP) level, a serum marker for inflammation. Patients with lower levels of CRP were found to be more likely to survive. Clinically, an increase in inflammatory markers, such as CRP and erythrocyte sedimentation rate, has been associated with poor outcomes in anti-cytotoxic T-lymphocyte-associated protein 4 (CTLA-4) antibody treatment.^34^ This was in line with the findings in our cohort receiving anti-PD-1/PD-L1 therapy. SHAP showed that lower levels of ALP influenced the model to predict better survival outcomes. Patients with advanced urothelial cancer, NSCLC, and melanoma can potentially develop bone metastasis and have a poor prognosis.^35^, ^36^, ^37^ ALP may be elevated in case of acute inflammation of the liver, cholecystitis, or as the result of bone diseases such as bone metastasis, and has been clinically associated with poor prognosis in different types of cancer.^38^, ^39^, ^40^, ^41^, ^42^, ^43^, ^44^

Patients with higher Hb levels were more likely to survive according to SHAP. In the literature, significantly lower concentrations of Hb were detected in patients with bladder cancer having bone metastasis, suggesting lower levels of Hb to be a risk factor for developing bone metastasis in newly diagnosed patients with bladder cancer.^42^ Furthermore, it was suggested that anemia may influence the treatment outcome, as it could correlate with tumor hypoxia which, in turn, could be associated with poor immunotherapy outcome.^45^, ^46^, ^47^ Increased neutrophil levels have been associated with decreased overall survival in patients treated with ipilimumab, while high lymphocyte counts upon anti-CTLA-4 blockade and higher levels of albumin following treatment with durvalumab have been associated with improved survival.^34^^,^^48^ These findings are in line with the SHAP explanations generated by the averaged predictions of our models.

SHAP plots split by tumor type identified the same features as important for the predictive model, albeit with slight differences in the order of importance. None of the laboratory features show any strong univariate positive or negative correlations with the survival outcome in our cohort; however, the most impactful features using SHAP explanations did show a slightly higher correlation compared with the other features (Supplementary Figures S6-S9, available at https://doi.org/10.1016/j.iotech.2024.100723).

Integration of information from different diagnostic data modalities provides an opportunity to objectively see the patient’s state from different perspectives, thus potentially developing better computer-aided diagnosis and prognosis systems.^9^^,^^18^^,^^49^ Overall survival is an endpoint that yields itself easily to integrated diagnostics, as different factors could simultaneously affect the survival of patients.^50^, ^51^, ^52^, ^53^, ^54^, ^55^, ^56^, ^57^ With respect to multimodal AI in immunotherapy, response assessment has also been a topic of research.^57^, ^58^, ^59^, ^60^, ^61^, ^62^ Response to immunotherapy in melanoma was predicted from complex biological data sources: T-cell receptor sequencing and the human leukocyte antigen.^58^ Johannet et al.^59^ predicted response in advanced melanoma by integrating histology specimens and clinical data, while pathology and genomic data were used alongside radiological images for response prediction in NSCLC in the study of Vanguri et al.^62^ The inclusion of histological data sources poses advantages by utilizing biological domain knowledge to help train an AI model. However, histology data are invasively obtained and fail to capture tumor heterogeneity due to sampling bias. By contrast, noninvasive diagnostic data, such as radiological imaging and blood-based laboratory tests, are routinely available during patient treatment and follow-up, and contain information about the overall status of the tumor and patient. These data sources have proven promising not only for response prediction in immunotherapy^60^^,^^61^^,^^63^ but also for monitoring clonal heterogeneity to help identify patients at risk of progression during treatment.^17^

Integration methodologies are mainly categorized in the literature into early, intermediate, and late fusion strategies.^10^^,^^14^ Multimodal data leveraging studies often use intermediate fusion strategies, utilizing joint feature-level learning for capturing concordant and/or complementary information across different data modalities during training. Real-world medical datasets are known to suffer from missing data and/or offer diagnostic information from different modalities that are often not perfectly aligned in time. These two drawbacks limit the size of datasets that could employ early and intermediate fusion strategies. We, therefore, opted for a late fusion approach, maximizing the usage of the available medical datasets of each modality during model training.

Past integrated diagnostics studies in immunotherapy used single time point data, making direct comparisons to our longitudinal study challenging. To our knowledge, this has been carried out in two previous studies in the context of NSCLC: mainly to predict RECIST response at 60 and 90 days^60^^,^^61^ and iRECIST-based progression-free survival (PFS) at 6 and 9 months.^63^ Similar to our study, the integration of laboratory data, CT scans, and clinical data was analyzed; however, additional clinical and/or genomic data were also included. The endpoint prediction task, the methods, and the size of the datasets in these studies were also different from ours. Both studies, similar to ours, reported an improved integrated performance with all the modalities compared with the performance of single modalities. The highest AUCs reported by Yang et al.^61^ were for the prediction of response at 90 days using pretreatment data (AUC_multimodal_: 0.80, AUC_radiomics_: 0.64, and AUC_blood_: 0.57). Our subanalysis of the NSCLC cohort for the prediction of overall survival at 1 year using pretreatment data showed AUC_multimodal_ of 0.71, AUC_CT_ of 0.62, and AUC_blood_ of 0.70 (Supplementary Table S4, available at https://doi.org/10.1016/j.iotech.2024.100723). Farina et al.^63^ reported AUC_multimodal_ of 0.824 and 0.753, AUC_CT_ of 0.740 and 0.702, and AUC_blood+clinical_ of 0.700 and 0.585 for PFS prediction at 6 and 9 months, respectively, using longitudinal data. Our analysis for 1-year survival prediction using longitudinal data showed an AUC_multimodal_ of 0.84, AUC_CT_ of 0.72, and AUC_blood_ of 0.81 in NSCLC. Our results of overall survival prediction at 1 year were higher than the results of PFS prediction at 9 months by Farina et al.^63^

Most integration studies show performance improvements in the integrated model compared with individual modality models.^50^^,^^53^^,^^54^ However, these studies integrated only two types of data. Studies that included more modalities for integration showed variable changes in the performance, based on which modalities were being combined.^52^^,^^55^ A similar trend was observed in our study with the inclusion of clinical parameters (Supplementary Table S2, available at https://doi.org/10.1016/j.iotech.2024.100723). Peisen et al.^57^ included tumor markers as part of their clinical data alongside patient demographics (age and sex) and radiomic features to predict response and survival in patients with advanced melanoma treated with immunotherapy. Their study showed an improved performance integrating radiomics and clinical data for survival prediction at 6 and 12 months, compared with clinical data alone. The drop in the prognostic performance when clinical parameters were added in some combinations in our study could probably be due to the limited number of the utilized clinical parameters.

Another limitation of our study is that we distinguished longitudinal examinations along the treatment timeline using a scalar value representing the positions of the examinations (in days), relative to the start of treatment, instead of utilizing specific methods for time-series data analysis. Subsequently, we computed the AUC on samples that were not fully independent. When accounting for this in a subanalysis (using only the latest examination per patient, Table 3), results showed a similar pattern to the rest of the analysis in terms of modest improvement in integrative performance.

Despite their promise, integration methods in healthcare have yet to demonstrate sufficient robustness for clinical usage. The field still requires extensive research to overcome the challenges that real-life medical datasets present to benefit from the complementary information that different diagnostic sources offer. Furthermore, validating the developed methods for generalizability across different centers, cancer types, treatments, diseases, and clinical outcomes is crucial for the clinical implementation of AI methods. An important requirement for integrational medical AI research is better collaboration between clinicians of different clinical specialties. As radiologists, pathologists, geneticists, and treating clinicians are each generating increasingly larger volumes of data per patient, hospitals need to account for the required digital infrastructure to efficiently store and make good use of this resource. European initiatives are now working on establishing cancer imaging repositories and as regulation begins to address the needs of medical AI practitioners, large-scale access to multicenter multimodal data will prove to be a paradigm shift for this subdomain of medical AI. Notwithstanding these challenges, the field of multimodal learning is rapidly evolving, with the potential to revolutionize the field of healthcare and personalized medicine.

## Conclusion

In this study, we used AI algorithms to predict the survival of patients with metastatic NSCLC, melanoma, and urothelial cancer treated with immunotherapy using multimodal diagnostic data. Our analysis showed a modest improvement in the prognostic performance integrating longitudinal CT imaging, blood-based laboratory data, and clinical parameters over the performance of the best-performing single modality, laboratory data. The improvement in the integrative performance was more prominent in the remaining modalities, CT, and clinical parameters, showing the potential of integrating different noninvasive diagnostic data. Future research will focus on implementing more sophisticated integration strategies to further improve the prognostic performance using AI in immunotherapy.

## Supplementary data

Supplementary Docx Supplementary Docx
