# Supplementary material for: Integrated noninvasive diagnostics for prediction of survival in immunotherapy
Source: Immunooncol Technol. 2024 Jul 9;24:100723. doi: 10.1016/j.iotech.2024.100723 (PMC11342748; doi:10.1016/j.iotech.2024.100723)
Supplement: Supplementary Docx [file mmc1.docx]

# **Supplementary Materials:**

## ***1. Methods***

## ***1.1. Data Preprocessing***

All the CT scans were cropped to only include the thoracic region using the method proposed by Zhang et al. [(1)](https://paperpile.com/c/tRJRjw/VAkh8). The scans were then resampled into 2 mm isotropic voxel size and cropped or padded to 164x218x218 voxels. Image intensities were clipped between -120 HU (fat) and +300 HU (trabecular bone) to enhance soft tissue. The resulting images were standardized and normalized to a 0-1 range. To embed a time dimension into the model, the intervals between the scan acquisition and the start of the treatment (in days) were also included as additional input features.

For the tabular blood-based laboratory data, rows and columns with ≥75% of missing values were discarded, decreasing the numbers of utilized blood markers from 66 to 33 markers (as detailed in section 1.2.2 and Supplementary Table T0). Non-numerical values, such as <mg, <mvg, <aggr, <Lip, or <Hem, were replaced by null, or lower or upper bound values when these values represented ranges, replacing <0.1, <0.3, <0.5, <1, <2, <3, <5 with 0 and >120 with 130. An iterative imputer with the Bayesian Ridge regression estimator [(2–5)](https://paperpile.com/c/tRJRjw/fV0FU+BxWmv+tDDLN+SDgOV) was used to estimate the remaining missing values in the laboratory data from the available features in the training sets of the 30 cross-validation folds. Supplementary Table T6 displays the percentages of imputed missing values. Finally, standardization of clinical and laboratory data features was also performed. Scikit-learn 0.24.1 was used for the implementation of the models [(6)](https://paperpile.com/c/tRJRjw/ANwSn).

## ***1.2. Single modality prognostic models***

### **1.2.1. Chest computed tomography imaging**

The preprocessed chest CT images were used to train a convolutional neural network (CNN) to predict the 1-year survival of the patients in a supervised manner. A 3D version of a ResNet18-like architecture [(7)](https://paperpile.com/c/tRJRjw/G0Nxa) was adopted, consisting of 20 convolutional layers, global average pooling, and four fully connected layers of sizes 256, 128, 64 and 1, respectively. The convolutional layers encoded each scan to 512 5x7x7-sized feature maps. The timespan between the acquisition of the scan and the start of the treatment was added as an additional input feature during training. The network was trained to minimize the focal loss using the Adam optimizer with cyclical learning rates within [1x 10^-5^ , 5x 10^-5^] [(8)](https://paperpile.com/c/tRJRjw/PRo20) with a batch size of either 3 or 6 scans in different MCCV splits, depending on the available GPUs. The parametric ReLU activation function, layer normalization and regularization (weight decay of 1 x 10^-3^, dropout and early stopping) were also employed. Keras 2.2.4 [(9)](https://paperpile.com/c/tRJRjw/dgdDG) and Tensorflow-gpu 1.12 [(10)](https://paperpile.com/c/tRJRjw/TBiYs) were used for the implementation.

### **1.2.2. Blood-based laboratory tests and clinical parameters**

The utilizedlood-based laboratory data consisted of longitudinal measurements of 33 laboratory parameters: hemoglobin (Hb), hematocrit (Ht), mean corpuscular volume (MCV), erythrocytes (RBC), thrombocytes (Plt), leukocytes (WBC), lymphocytes (Lympho), monocytes (Mono), eosinophils (Eos), basophils (Baso), neutrophils (Neutr), a combination of neutrophils, basophils and eosinophils (NeutrGran), immature granulocytes (ImmGran), erythrocyte sedimentation rate (ESR), C-reactive protein (CRP), total bilirubin (TBIL), direct bilirubin (DBIL), alkaline phosphatase (ALP), aspartate aminotransferase (AST), alanine aminotransferase (ALT), creatinine (Cr), glomerular filtration rate (GFR), sodium, potassium, chloride, bicarbonate, phosphate, calcium, magnesium, urea, glucose, total protein, and albumin.

The timespan between the examination and start of treatment (SoT) was also added as an input feature. Clinical parameters consisted of three features: age, sex, and tumor type. These features were defined at the start of the treatment and are not longitudinal.

Random forest (RF) [(11)](https://paperpile.com/c/tRJRjw/MQs3G) models were employed to predict survival at 1 year using laboratory data, while clinical data was trained to predict the same endpoint using support vector machines (SVM) [(12)](https://paperpile.com/c/tRJRjw/ZEsMw). Hyperparameter optimization was performed in both types of analysis, and the models with the best performance on the validation set were selected for test set evaluation. Scikit-learn 0.24.1 [(6)](https://paperpile.com/c/tRJRjw/ANwSn) was used for the implementation.

## ***2. Supplementary Figures:***


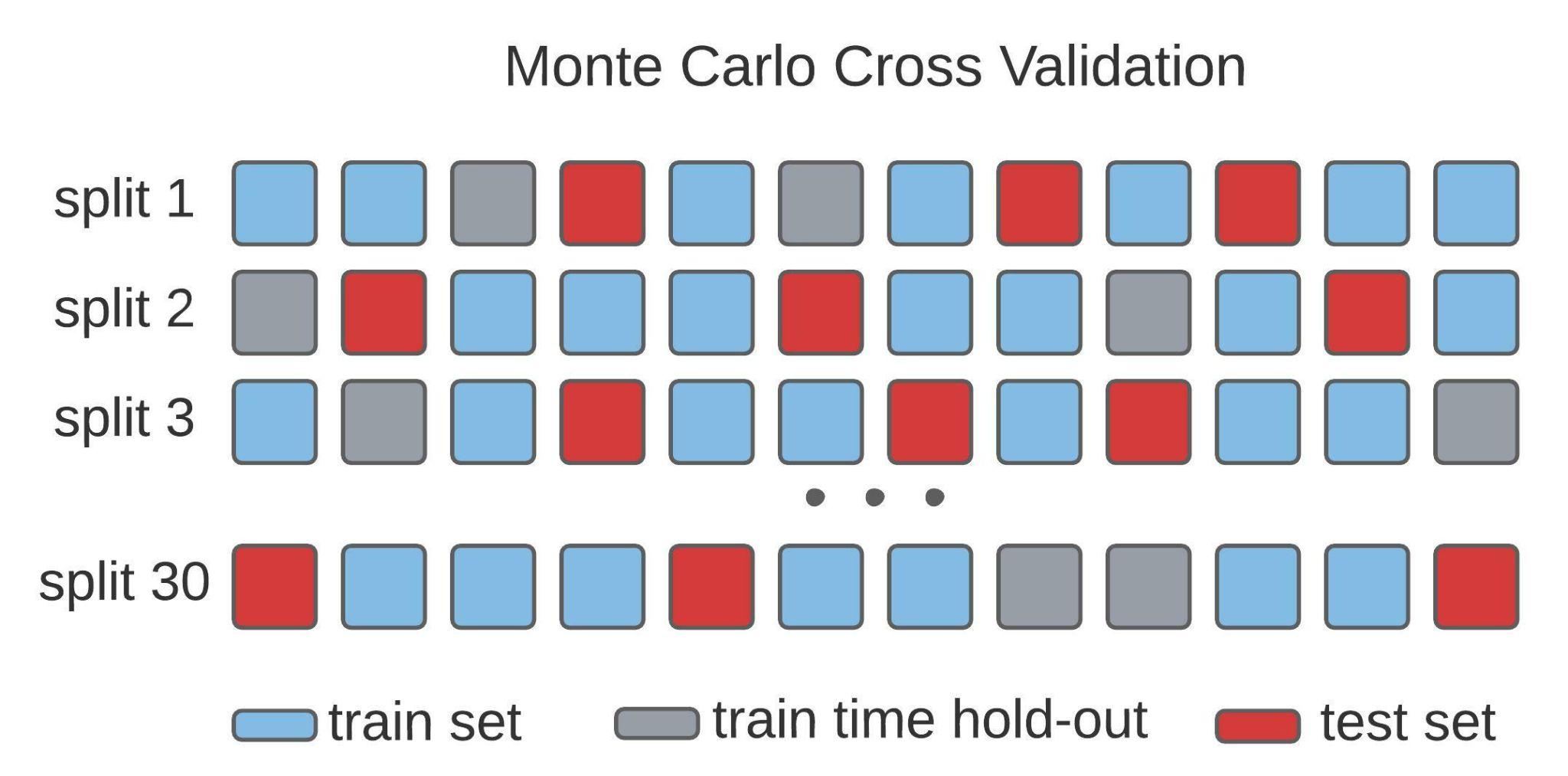


Figure S1. Monte Carlo cross validation (MCCV)


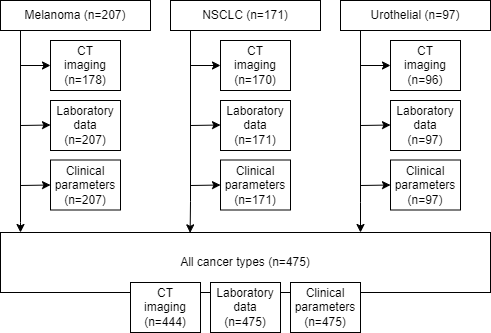


Figure S2. A schematic diagram visualizing the included patients


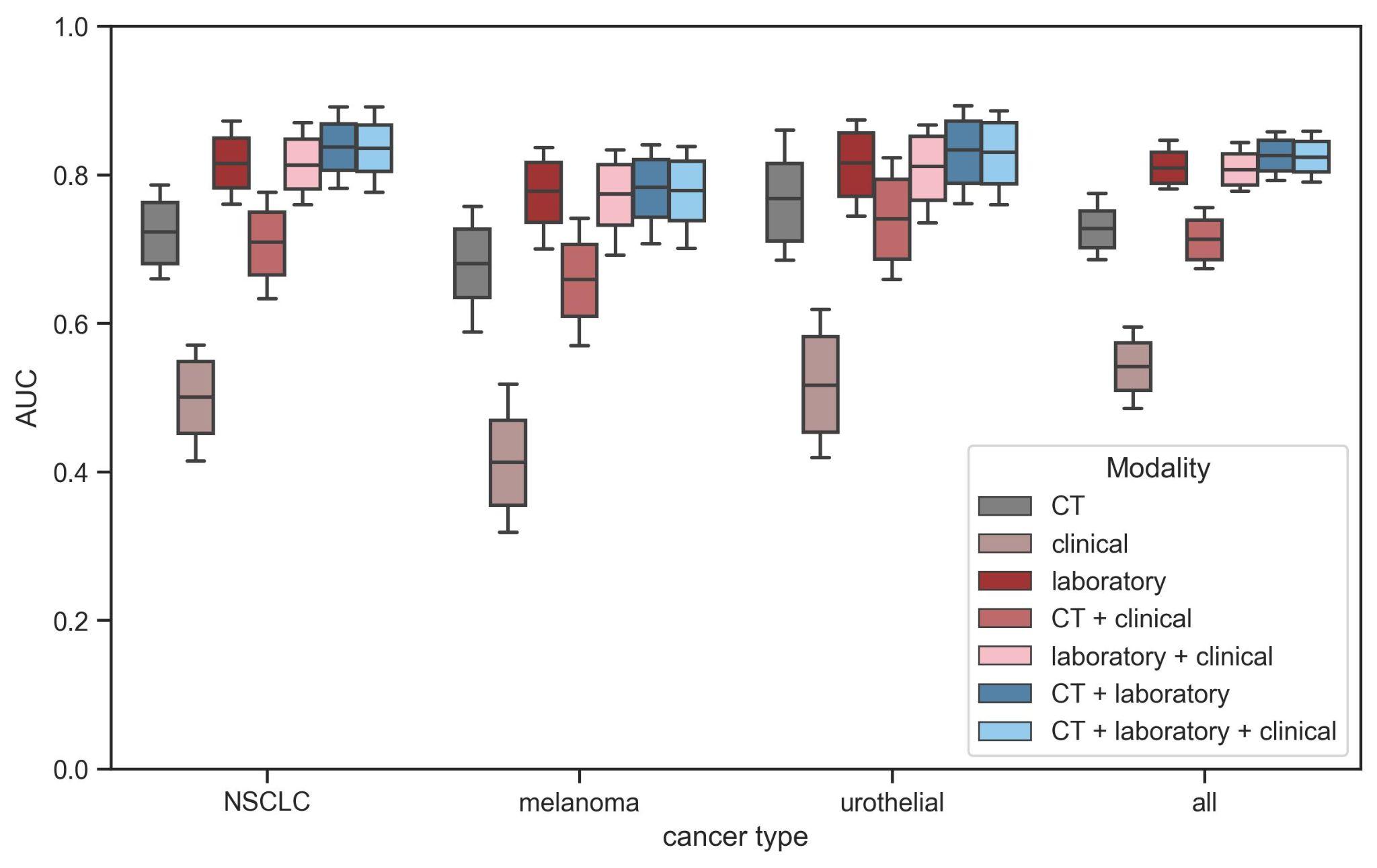
Figure S3. Survival classification performance evaluation based on cancer type and different combinations of modalities


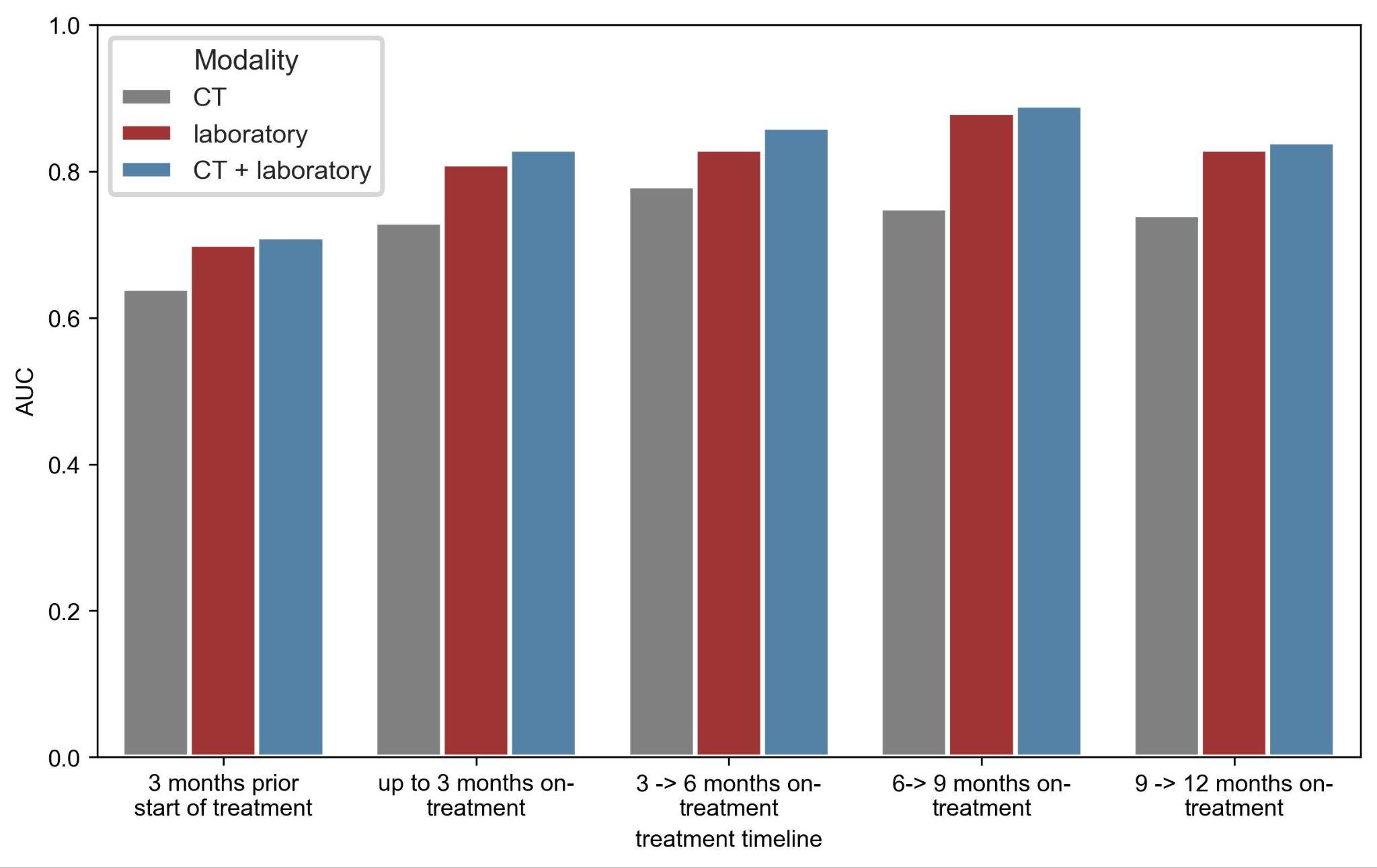


Figure S4. Survival classification performance in 3-month intervals pre and on-treatment data using longitudinal modalities


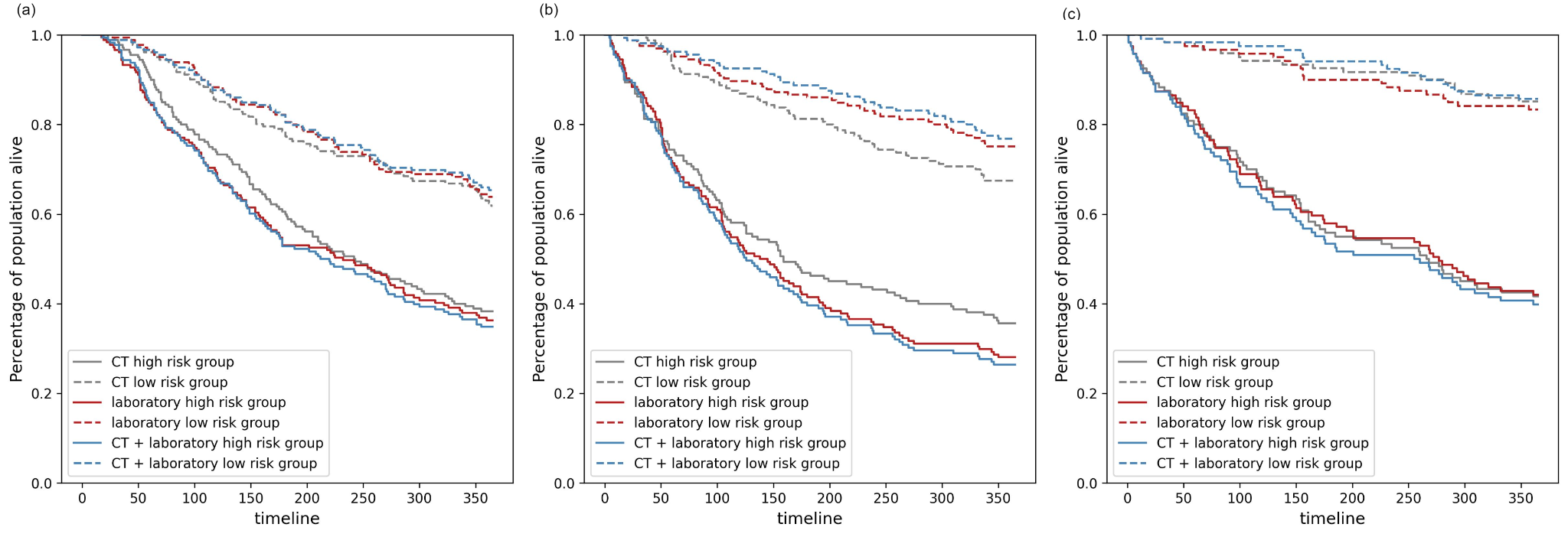


Figure S5. Kaplan-Meier (KM) survival curves for high and low-risk groups of patients using CT, laboratory and CT-laboratory combined modalities, using the latest exam per patient (a) pre-treatment data (b) on-treatment data acquired up to three months and (c) six months.


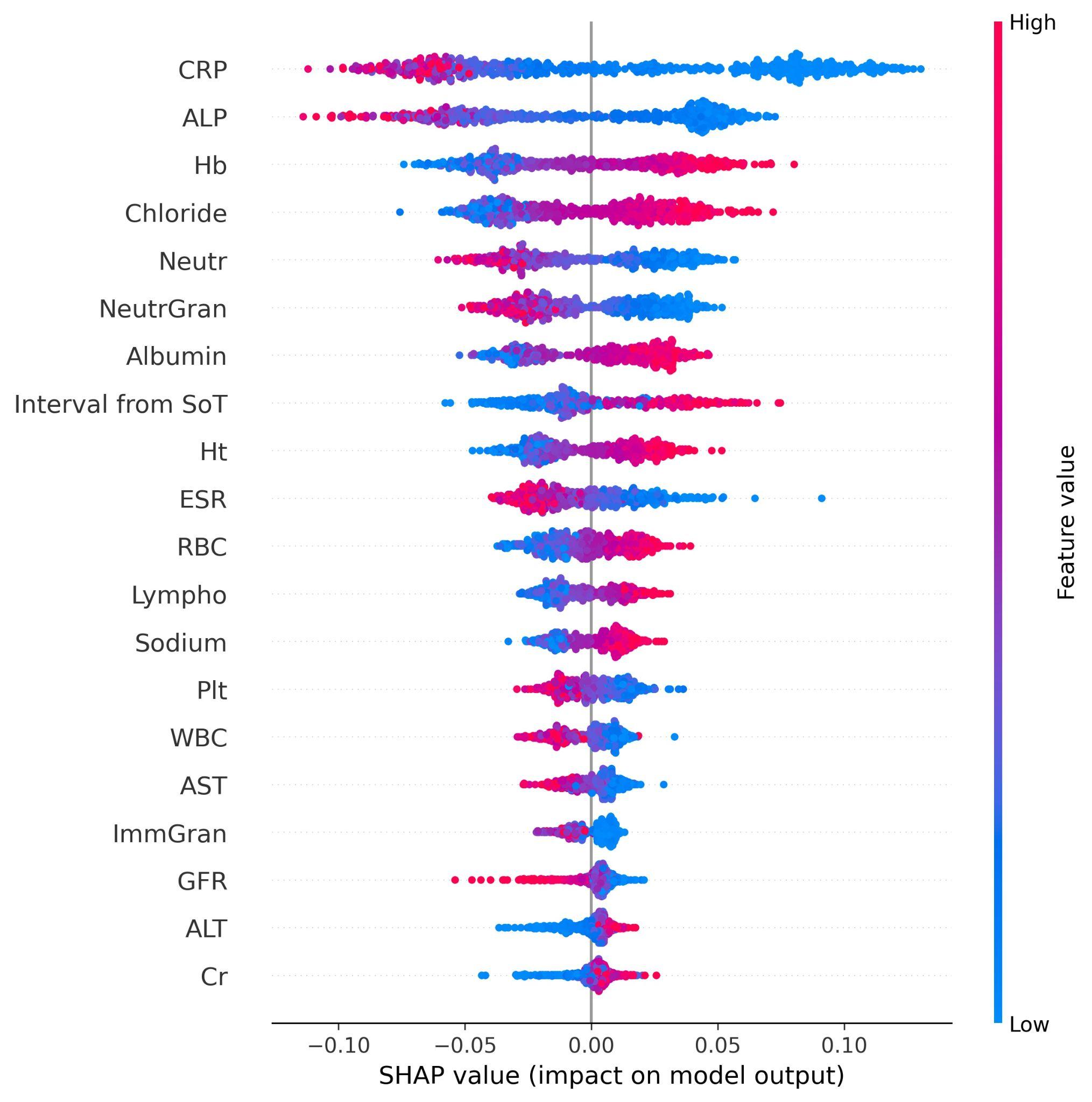


Figure S6. Interpretability of the Random Forest model trained with laboratory data using SHAP explanations on the NSCLC cancer subgroup


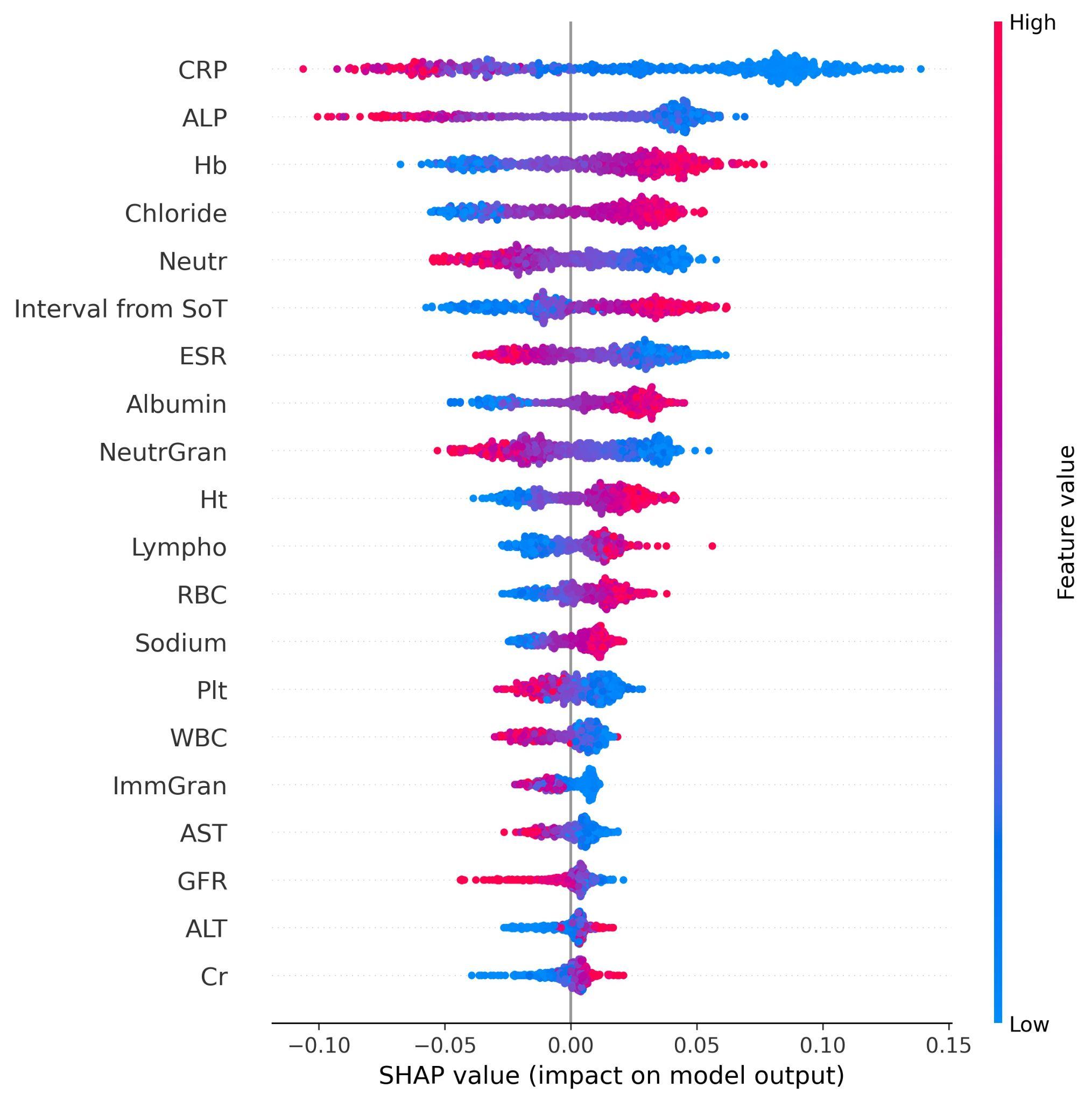


Figure S7. Interpretability of the Random Forest model trained with laboratory data using SHAP explanations on the melanoma subgroup


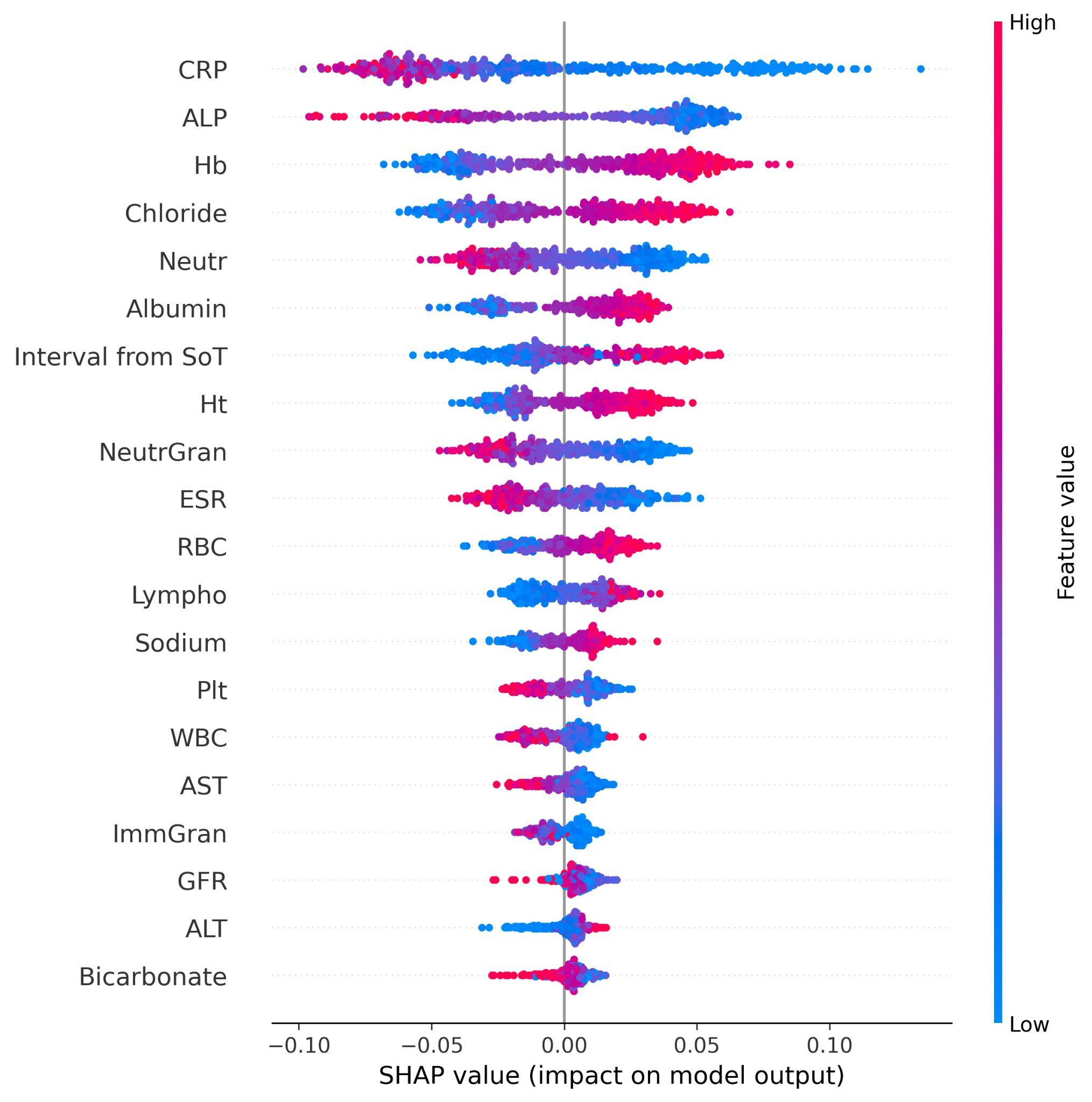


Figure S8. Interpretability of the Random Forest model trained with laboratory data using SHAP explanations on the urothelial cancer subgroup


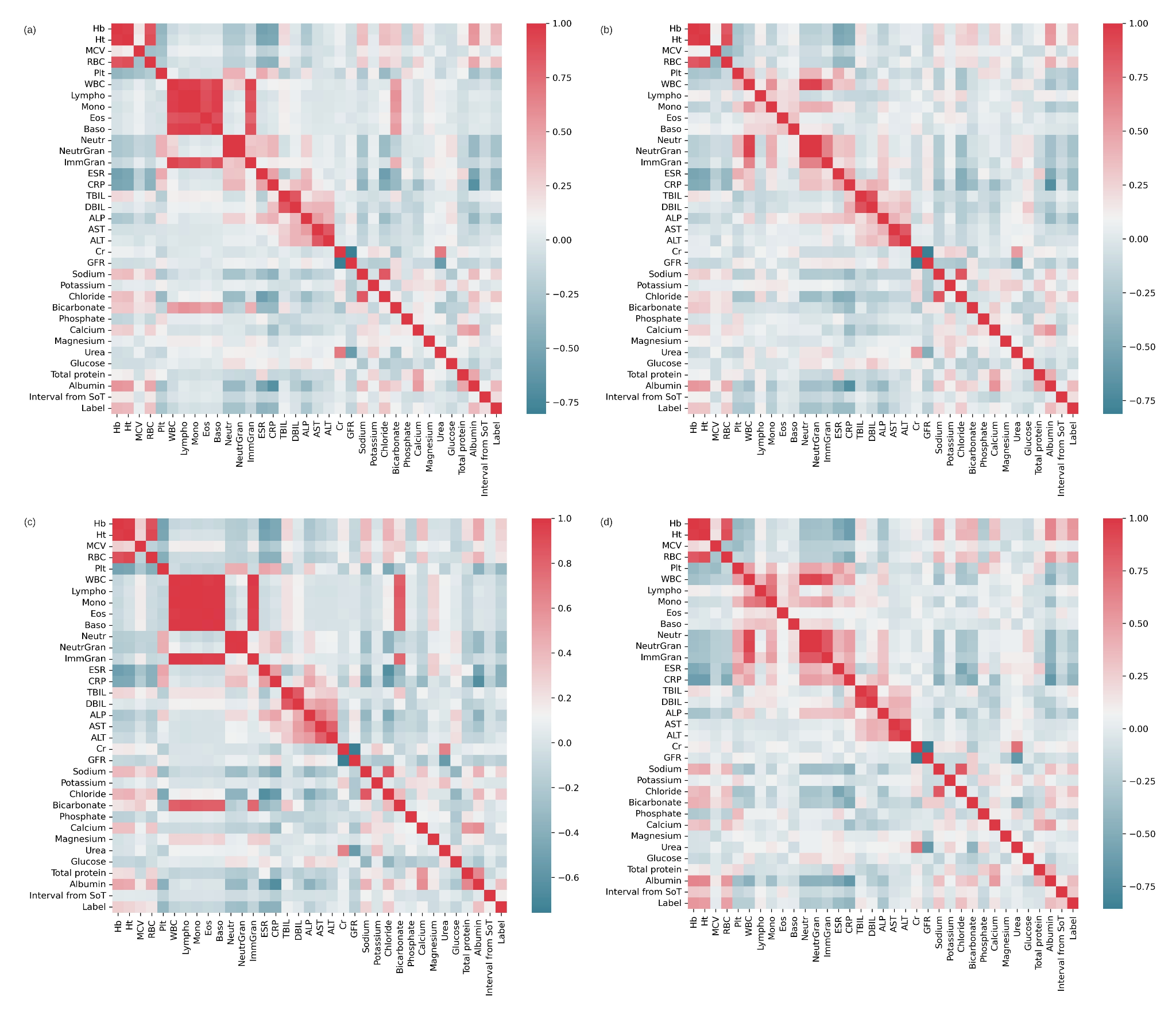


Figure S9. The pairwise correlation of laboratory data features for (a) all, (b) NSCLC, (c) melanoma, and (d) urothelial cancer types

## ***3. Supplementary Tables:***

Table S1: A list of utilized routine blood markers and their abbreviations

| Blood marker | Abbreviation | Unit |
| --- | --- | --- |
| hemoglobin | Hb | mmol/l |
| hematocrit | Ht | l/l |
| mean corpuscular volume | MCV | (Ht/Rbc) fl |
| erythrocytes | RBC | 10E12/l |
| thrombocytes | Plt | 10E9/l |
| leukocytes | WBC | 10E9/l |
| lymphocytes | Lympho | % |
| monocytes | Mono | 10E9/l |
| eosinophils | Eos | % |
| basophils | Baso | % |
| neutrophils | Neutr | 10E9/l |
| a combination of neutrophils, basophils and eosinophils | NeutrGran | 10E9/l |
| immature granulocytes | ImmGran | 10E9/l |
| erythrocyte sedimentation rate | ESR | mm/uur |
| C-reactive protein | CRP | mg/l |
| total bilirubin | TBIL | µmol/l |
| direct bilirubin | DBIL | µmol/l |
| alkaline phosphatase | ALP | U/l |
| aspartate aminotransferase | AST | U/l |
| alanine aminotransferase | ALT | U/l |
| creatinine | Cr | µmol/l |
| glomerular filtration rate | GFR | ml/min/1.73m2 |
| sodium | - | mmol/l |
| potassium | - | mmol/l |
| chloride | - | mmol/l |
| bicarbonate | - | mmol/l |
| phosphate | - | mmol/l |
| calcium | - | mmol/l |
| magnesium | - | mmol/l |
| urea | - | mmol/l |
| glucose | - | mmol/l |
| total protein | - | g/l |
| albumin | - | g/l |

Table S2. Prognostic performance of all combinations of modalities

| Modality | (N survival, N death) | | AUC  (95% CI) | Sensitivity  (95% CI) | Specificity  (95% CI) | PPV  (95% CI) | NPV  (95% CI) | p-value |
| --- | --- | --- | --- | --- | --- | --- | --- | --- |
| CT | (942, 617) | | 0.73  (0.70- 0.75) | 0.60  (0.57- 0.63) | 0.75  (0.71- 0.78) | 0.79  (0.75- 0.81) | 0.55  (0.52- 0.58) | <0.001 |
| Laboratory | (942, 617) | | 0.81  (0.79- 0.83) | 0.68  (0.65- 0.71) | 0.78  (0.75- 0.81) | 0.83  (0.80- 0.85) | 0.61  (0.58- 0.65) | <0.001 |
| Clinical | (1099, 460) | | 0.54  (0.51- 0.57) | 0.52  (0.49- 0.55) | 0.55  (0.50- 0.59) | 0.73  (0.70- 0.77) | 0.32  (0.29- 0.36) | 0.010 |
| CT + clinical | (942, 617) | | 0.71  (0.69- 0.74) | 0.60  (0.57- 0.63) | 0.72  (0.69- 0.76) | 0.77  (0.74- 0.80) | 0.54  (0.51- 0.58) | <0.001 |
| Laboratory + clinical | (942, 617) | | 0.81  (0.79- 0.83) | 0.67  (0.64- 0.70) | 0.78  (0.75- 0.81) | 0.82  (0.80- 0.85) | 0.61  (0.57- 0.64) | <0.001 |
| CT + laboratory | (942, 617) | | **0.83**  **(0.81- 0.85)** | 0.67  (0.64- 0.70) | 0.79  (0.76- 0.82) | 0.83  (0.81- 0.86) | 0.61  (0.58- 0.64) | <0.001 |
| CT + laboratory + clinical | (942, 617) | | **0.82**  **(0.80- 0.84)** | **0.68**  **(0.65-0.70)** | **0.80**  **(0.77- 0.83)** | **0.84**  **(0.81- 0.86)** | **0.62**  **(0.58- 0.65)** | **<0.001** |

N indicates the number of examinations

Table S3. Prognostic performance of all combinations of modalities stratified by cancer type

| Modality | AUC (95% CI) | | | |
| --- | --- | --- | --- | --- |
|  | NSCLC | Melanoma | Urothelial | All combined |
| N | 587 | 580 | 392 | 1559 |
| CT | 0.72  (0.68- 0.76) | 0.68  (0.63- 0.73) | 0.77  (0.71- 0.81) | 0.73  (0.70- 0.75) |
| Laboratory | 0.81  (0.78- 0.85) | 0.78  (0.74- 0.82) | 0.82  (0.77- 0.86) | 0.81  (0.79- 0.83) |
| Clinical | 0.50*  (0.45- 0.55) | 0.41  (0.36- 0.47) | 0.52*  (0.45- 0.58) | 0.54  (0.51- 0.57) |
| CT + clinical | 0.71  (0.67- 0.75) | 0.66  (0.61- 0.71) | 0.74  (0.69- 0.79) | 0.71  (0.69- 0.74) |
| Laboratory + clinical | 0.81  (0.78- 0.85) | 0.77  (0.73- 0.81) | 0.81  (0.77- 0.85) | 0.81  (0.79- 0.83) |
| CT + laboratory | 0.84  (0.81- 0.87) | 0.78  (0.74- 0.82) | 0.83  (0.79- 0.87) | 0.83  (0.80- 0.85) |
| **CT + laboratory + clinical** | **0.84**  **(0.80- 0.87)** | **0.78**  **(0.74- 0.82)** | **0.83**  **(0.79- 0.87)** | **0.82**  **(0.80- 0.85)** |

*p*<0.05 in all experiments, except the ones followed with an *. N indicates the number of examinations

Table S4. Prognostic performance of all combinations of modalities using pre-treatment data for each cancer type

| Modality | AUC (95% CI) | | | |
| --- | --- | --- | --- | --- |
|  | NSCLC | Melanoma | Urothelial | All combined |
| N | 185 | 146 | 133 | 464 |
| CT | 0.62  (0.53- 0.70) | 0.60  (0.49- 0.70) | 0.69  (0.59- 0.77) | 0.65  (0.60- 0.70) |
| Laboratory | 0.70  (0.62- 0.77) | 0.70  (0.60- 0.78) | 0.64  (0.55- 0.74) | 0.69  (0.65- 0.74) |
| Clinical | 0.51*  (0.42- 0.60) | 0.42*  (0.31- 0.53) | 0.52*  (0.43- 0.62) | 0.56  (0.51- 0.61) |
| CT + clinical | 0.62  (0.53- 0.69) | 0.57*  (0.46- 0.67) | 0.69  (0.60- 0.78) | 0.65  (0.60- 0.70) |
| Laboratory + clinical | 0.70  (0.61- 0.77) | 0.69  (0.60- 0.77) | 0.64  (0.55- 0.74) | 0.70  (0.65- 0.74) |
| CT + laboratory | 0.71  (0.63- 0.78) | 0.70  (0.61- 0.78) | 0.67  (0.58- 0.77) | 0.71  (0.66- 0.75) |
| CT + laboratory + clinical | **0.71**  **(0.63- 0.78)** | **0.69**  **(0.59- 0.76)** | **0.68**  **(0.58- 0.77)** | **0.71**  **(0.66- 0.75)** |

*p*<0.05 in all experiments, except the ones followed with an *. N indicates the number of examinations

Table S5. The difference of median survival time in low and high-risk groups in KM analysis for pre-treatment, early and late on-treatment data

|  | Modality | Pre-treatment | On-treatment | |
| --- | --- | --- | --- | --- |
|  |  | -92 - 0 days | 0 - 92 days | 92 - 183 days |
| Difference in median survival time | CT | 123 | 206 | 99 |
|  | Laboratory | 133 | 227 | 88 |
|  | Integrated | 153 | 239 | 110 |
| *p*-value* | All experiments | <0.001 | <0.001 | <0.001 |

* The statistical significance between high and low risk groups within each modality.

The pre-treatment data included the latest exam (pair) per patient acquired within the last 3 months before start of treatment. The on-treatment data included the latest exam acquired between start of treatment and 3 months, and between 3 months and 6 months.

Table S6. The prognostic performance of longitudinal data modalities and their integration in three-month intervals, using all data samples.

|  | Modality | Pre-treatment | On-treatment | | | |
| --- | --- | --- | --- | --- | --- | --- |
|  |  | <= 0 days | 0 - 92 days | 92 - 184 days | 184 - 276 days | 276 - 365 days |
| AUC  (95% CI) | CT | 0.66  (0.61- 0.71) | 0.72  (0.67- 0.77) | 0.78  (0.73- 0.83) | 0.78  (0.70- 0.85) | 0.72  (0.62- 0.81) |
|  | Laboratory | 0.69  (0.64- 0.74) | 0.78  (0.74- 0.82) | 0.83  (0.77- 0.87) | 0.88  (0.83- 0.93) | 0.82  (0.74- 0.89) |
|  | Integrated | **0.71**  **(0.66- 0.75)** | **0.81**  **(0.77- 0.85)** | **0.86**  **(0.80- 0.90)** | **0.88**  **(0.81- 0.93)** | **0.83**  **(0.76- 0.90)** |
| p-value | All experiments | <0.001 | <0.001 | <0.001 | <0.001 | <0.001 |

Table S7: The percentages of imputed missing values in both laboratory blood markers and paired CT and blood markers datasets

| Marker | Laboratory data | Paired CT and laboratory data |
| --- | --- | --- |
| Hb | 2.88 | 1.2 |
| Cr | 3.07 | 1.69 |
| GFR | 3.07 | 1.69 |
| WBC | 4.33 | 1.99 |
| Plt | 4.43 | 1.99 |
| Ht | 4.61 | 2.83 |
| ASAT | 10.56 | 4.33 |
| ALAT | 10.58 | 4.39 |
| ALP | 10.66 | 4.39 |
| potassium | 14.02 | 13.85 |
| sodium | 14.08 | 13.85 |
| RBC | 14.16 | 11.74 |
| MCV | 15.22 | 13.25 |
| TBIL | 16.06 | 11.08 |
| albumin | 17.74 | 15.83 |
| Ca | 19.09 | 16.62 |
| NeutrGran | 24.6 | 19.63 |
| phosphate | 29.57 | 35.16 |
| CRP | 31.8 | 36.06 |
| total protein | 32.04 | 28.3 |
| ureum | 35.64 | 31.73 |
| Neutr | 36.78 | 32.03 |
| Mono | 36.91 | 32.03 |
| Lympho | 36.92 | 32.03 |
| Eos | 36.92 | 32.03 |
| Baso | 36.92 | 32.03 |
| ImmGran | 36.94 | 32.03 |
| glucose | 38.05 | 32.21 |
| magnesium | 48.05 | 42.2 |
| DBIL | 60.32 | 66.47 |
| chloride | 65.41 | 61.17 |
| bicarbonate | 65.74 | 61.23 |
| ESR | 67.45 | 71.22 |

**References:**

1. [Zhang P, Wang F, Zheng Y. Self supervised deep representation learning for fine-grained body part recognition. In: Proceedings - International Symposium on Biomedical Imaging. IEEE Computer Society; 2017. p. 578–82.](http://paperpile.com/b/tRJRjw/VAkh8)

2. [Buck SF. A Method of Estimation of Missing Values in Multivariate Data Suitable for use with an Electronic Computer. J R Stat Soc Series B Stat Methodol. 1960;22(2):302–6.](http://paperpile.com/b/tRJRjw/fV0FU)

3. [van Buuren S, Groothuis-Oudshoorn K. mice: Multivariate Imputation by Chained Equations in R. J Stat Softw. 2011 Dec 12;45:1–67.](http://paperpile.com/b/tRJRjw/BxWmv)

4. [MacKay DJC. Bayesian interpolation. Neural Comput. 1992 May;4(3):415–47.](http://paperpile.com/b/tRJRjw/tDDLN)

5. [Tipping ME. 10.1162/15324430152748236. Appl Phys Lett [Internet]. 2000;1. Available from:](http://paperpile.com/b/tRJRjw/SDgOV) <https://doi.org/10.1162/15324430152748236>

6. [Pedregosa F, Varoquaux G, Gramfort A, Michel V, Thirion B, Grisel O, et al. Scikit-learn: Machine learning in Python. the Journal of machine Learning research. 2011;12:2825–30.](http://paperpile.com/b/tRJRjw/ANwSn)

7. [He K, Zhang X, Ren S, Sun J. Deep Residual Learning for Image Recognition. 2015 Dec 10; Available from:](http://paperpile.com/b/tRJRjw/G0Nxa) <http://arxiv.org/abs/1512.03385>

8. [Smith LN. Cyclical learning rates for training neural networks. In: 2017 IEEE Winter Conference on Applications of Computer Vision (WACV) [Internet]. IEEE; 2017. Available from:](http://paperpile.com/b/tRJRjw/PRo20) <http://dx.doi.org/10.1109/wacv.2017.58>

9. [Chollet F, Others. Keras [Internet]. 2015. Available from:](http://paperpile.com/b/tRJRjw/dgdDG) <https://keras.io>

10. [Abadi M, Agarwal A, Barham P, Brevdo E, Chen Z, Citro C, et al. TensorFlow: Large-Scale Machine Learning on Heterogeneous Distributed Systems [Internet]. arXiv [cs.DC]. 2016. Available from:](http://paperpile.com/b/tRJRjw/TBiYs) <http://arxiv.org/abs/1603.04467>

11. [Breiman L. Random Forests. Mach Learn. 2001 Oct 1;45(1):5–32.](http://paperpile.com/b/tRJRjw/MQs3G)

12. [Hearst MA, Dumais ST, Osuna E, Platt J, Scholkopf B. Support vector machines. IEEE Intelligent Systems and their Applications. 1998 Jul;13(4):18–28.](http://paperpile.com/b/tRJRjw/ZEsMw)
